# Extended disordered regions of ribosome-associated NAC proteins paralogs belong only to the germline in *Drosophila melanogaster*

**DOI:** 10.1038/s41598-022-15233-3

**Published:** 2022-07-01

**Authors:** Galina L. Kogan, Elena A. Mikhaleva, Oxana M. Olenkina, Sergei S. Ryazansky, Oxana V. Galzitskaya, Yuri A. Abramov, Toomas A. Leinsoo, Natalia V. Akulenko, Sergey A. Lavrov, Vladimir A. Gvozdev

**Affiliations:** 1 NRC “Kurchatov Institute”-Institute of Molecular Genetics, 123182 Moscow, Russia; 2 Institute of Protein Research, Russian Academy of Sciences, 142290 Pushchino, Russia; 3 Institute of Theoretical and Experimental Biophysics, Russian Academy of Sciences, 142290 Pushchino, Russia

**Keywords:** Cell biology, Developmental biology

## Abstract

The nascent polypeptide-associated complex (NAC) consisting of α- and β-subunits is an essential ribosome-associated protein conserved in eukaryotes. NAC is a ubiquitously expressed co-translational regulator of nascent protein folding and sorting providing for homeostasis of cellular proteins. Here we report on discovering the germline-specific NACαβ paralogs (gNACs), whose β-subunits, non-distinguishable by ordinary immunodetection, are encoded by five highly homologous gene copies, while the α-subunit is encoded by a single *αNAC* gene. The gNAC expression is detected in the primordial embryonic and adult gonads via immunostaining. The germline-specific α and β subunits differ from the ubiquitously expressed paralogs by the extended intrinsically disordered regions (IDRs) acquired at the N- and C-termini of the coding regions, predicted to be phosphorylated. The presence of distinct phosphorylated isoforms of gNAC-β subunits is confirmed by comparing of their profiles by 2D-isoeletrofocusing resolution before and after phosphatase treatment of testis ribosomes. We revealed that the predicted S/T sites of phosphorylation in the individual orthologous IDRs of gNAC-β sequences of *Drosophila* species are positionally conserved despite these disordered regions are drastically different. We propose the IDR-dependent molecular crowding and specific coordination of NAC and other proteostasis regulatory factors at the ribosomes of germinal cells. Our findings imply that there may be a functional crosstalk between the germinal and ubiquitous α- and β-subunits based on assessing their depletion effects on the fly viability and gonad development.

## Introduction

The evolutionary conservative NAC (nascent polypeptide associated complex consisting of α and β subunits) interacts with translating ribosomes and functions as a ubiquitous ATP-independent chaperone in eukaryotes^1,2^. It is suggested that the majority of translating ribosomes are associated with NAC^3,4^. NAC is involved in co-translational protein folding^5^ and delivery of mitochondrial precursor proteins^6^. Recently, new mechanistic insights were achieved in the studies of NAC-β subunit function by the demonstration that N-terminal of NAC-β subunit is able to be inserted deeply into the ribosomal tunnel, close to polypeptide transferase center, to sense nascent polypeptide^7^. The sensing NAC-β ability is coupled with its flexibility and conformational switches that either activate or prevent its alternative associations with the translocon complex of the endoplasmic reticulum (ER) or signal recognizing particle (SRP). These conformational changes of flexible NAC structure are suggested to regulate correct protein sorting for membrane components or secretion. NAC modulates protein transmission to ER lumen or cytosol, ensuring a specific co-translational protein biogenesis pathway^1–3,8^.

Here we have focused on the specific paralogs of NAC-β and NAC-α proteins expressed in the germ cells in *D. melanogaster*. Earlier, for the first time to our knowledge, we have discovered the expression of tissue specific NAC subunit in eukaryotes, detecting testis-specific amplified *βNAC* paralogs designated as the *βNACtes* genes (see FlyBase.org data and^9^). The amplified *βNACtes* genes were found in the genomes of sister species, *D*. *melanogaster* and *D*. *simulans,* but not in the closely related *D*. *yakuba*^9^. The generation of rat anti-NACtes Abs allowed us to reveal exclusively germinal, mostly spermatocyte expression of the *βNACtes* genes^10^. Here we further extended these studies by detecting the germinal NAC-α subunit as a product of the unique gene *CG4415* in the *D. melanogaster* genome thus proving the existence of the germline specific heterodimeric NAC-αβ, gNACs. We also showed that both germinal α- and β-subunits differ from ubiquitously expressed paralogs by the acquirement of extensive intrinsically disordered regions (IDRs), primarily at the N- and C-ends, respectively. The IDRs are known to be important chaperone functional regions^11,12^ and their significant length increase in gNAC may indicate a complex role for IDRs in the detection of conformational switches of clients in germ cells. Albeit functions of gNAC are still mysterious, it is believed that acquired extended IDRs are generally prone to extensive post-translational modifications (PTMs)^13^ as well as to the implementation of protein–protein interactions^14^. IDRs include various PTM sites involved in different regulatory pathways including cell fate and differentiation determination^15^ and characterized be the accelerated evolutionary diversification^16^. Disruption of disordered regions interactions ensuring protein–protein communications was shown to be resulted in the profound disturbances of regulatory processes^17^.

The germinal βNAC subunit was shown to be accumulated in ribosomes^10^. The 2D-electrophoresis with isoelectric focusing of testis ribosomal extract followed by specific immunostaining revealed a set of resolved spots of gNAC-β isoforms. The phosphatase treatment diminished the negative charges of these isoforms, indicating the presence of predicted gNAC-β phosphorylation.

## Results

### Detection of the germ cell-specific α- and β-paralogs forming the heterodimeric germinal NAC

**Figure 1:**
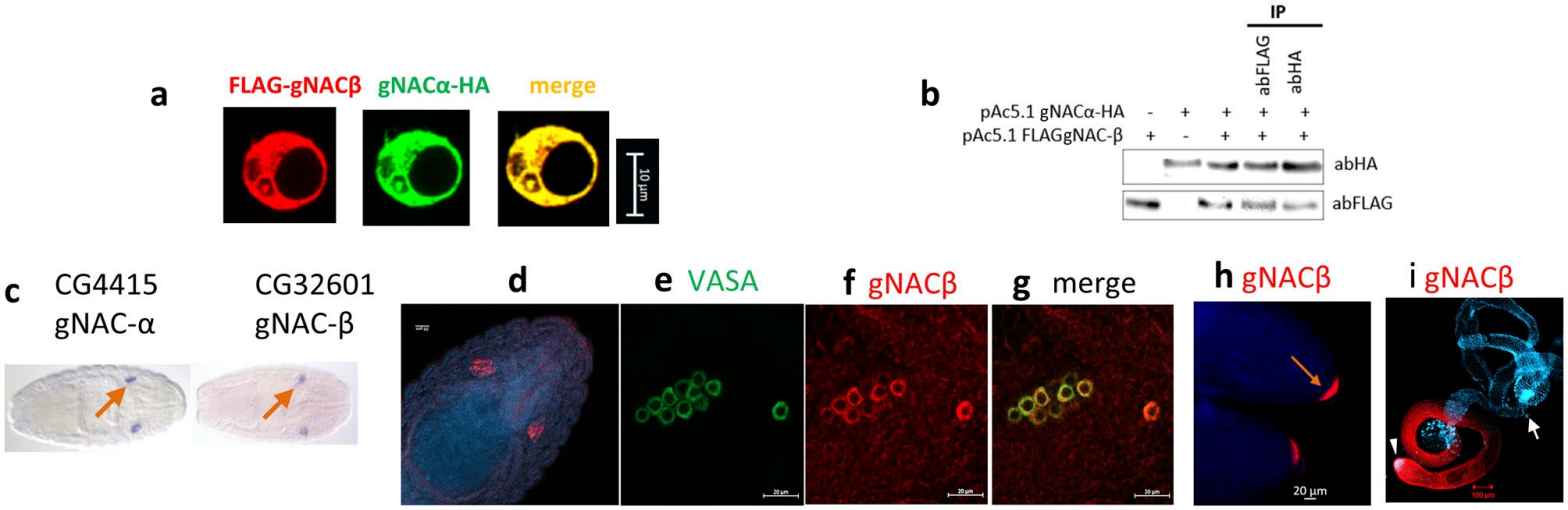
Detection of gNAC-α/gNAC-β heterodimer in S2 cells and gNAC-β in embryos. (**a**) Transfecting S2 cells to express pAc5.1 gNAC-αHA and pAc5.1 FLAGgNAC-β plasmids encoding the corresponding NAC subunits. (**b**) Generation and detection of gNAC after Western-blot analysis of heterodimers immunoprecipitated by abFLAG and abHA and detected using the same abs. (**c**) FISH-hybridization to detect *CG4415* and *CG32601* genes expression in the 14th stage embryos (from BDGP in situ homepage, https://insitu.fruitfly.org). (**d**) gNACβ immunostaining of primordial gonads; (**e–g**) migrating primordial gonadal cells, VASA (germ cell marker) and gNACβ colocalization. (**h**) Oocytes, late stages of oogenesis, gNAC-β accumulation at the posterior poles of oocytes (arrow). (**i**) Strong staining of testis spermatocyte, gNAC-unstained somatic accessory gland (DAPI stained, arrow) and testis tip (arrowhead). Original blots for (**b**) in Expanded file 1 in Supplementary.

Earlier, we have annotated five testis expressed paralogs of the ubiquitously expressed β-subunit of the *NAC* (*bic*) gene^9^. These paralogs are represented by two pairs of adjacent genes in the 12E8-9 region of X-chromosome of *D. melanogaster* (Flybase.org and^18,19^) and a single gene outside the 12E2 region^18,20^. The gene *CG4415* (located at the tip of 2L chromosome, the 21E3 region) encodes α-NAC subunit and was shown to be expressed in the embryonic gonads (Fisher et al. 2012, Flybase^19^). Moreover, a network analysis of protein–protein interactions using the STRING database (v. 10.5)^21^ (https://string-db.org/) indicated that NAC-βtes4 protein can possibly interact with *CG4415* gene product (NAC-α subunit). To directly demonstrate the heterodimerization of the putative germinal α-NAC and β-NACtes subunits, we performed co-transfection of S2 somatic *D. melanogaster* cells lacking the expression of germinal β-subunit with two plasmids encoding the Flag-tagged gNAC-β (NAC-βtes4) and HA-tagged gNAC-α subunits. The complete colocalization of FLAG-gNAC-β and HA-gNAC-α proteins point to the formation of germinal gNACαβ-heterodimer (Fig. 1a). The putative heterodimer was immunoprecipitated using anti-FLAG or anti-HA Abs and subsequent WB-analysis confirmed the predicted interaction of FLAG-gNAC-β and HA-gNAC-α subunits (Fig. 1b).

The *CG4415* gene transcripts were detected in the embryonic primordial gonads by in situ hybridization (Fig. 1c, from BDGP in situ homepage), and we revealed the presence of the βNACtes protein in the germline tissues of embryos by immunostaining with βNACtes Abs (Fig. 1d–i). The gNAC-α mRNA and βNACtes subunits similarly distributed in the primordial embryonic gonad (Fig. 1c,d) allowed us to further use the gNAC designation for heterodimeric germline specific NAC proteins, containing a single definite germinal NAC-β subunit, earlier termed as βNACtes^10^ and the newly detected gNAC-α subunit. The clusters of embryonic germinal cells migrating to primordial somatic gonadal cells were detected using immunostaining for the germinal VASA marker and the gNAC-β (Fig. 1e–g). In accordance with our earlier observation^10^, we found the strong gNAC-β expression in testis spermatocytes, but not in somatic testis accessory glands (Fig. 1h, arrow) as well as gNAC-β accumulation at the late stage of oogenesis in the germ plasm at the posterior pole of the mature egg (Fig. 1i)^22,23^.

### The germinal α- and β-subunits are enriched with IDRs

**Figure 2:**
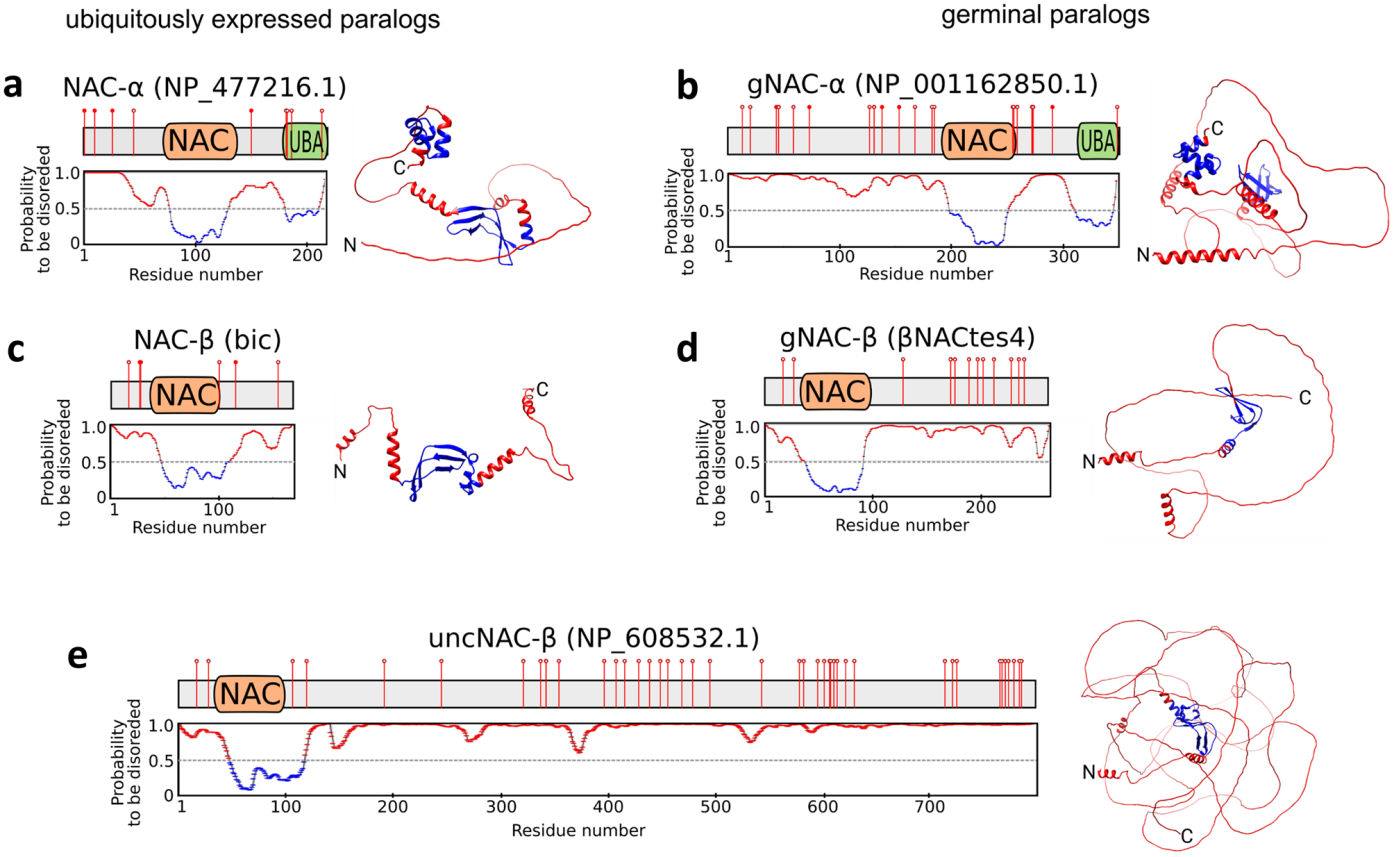
Order–disorder profiles of NAC containing polypeptides with corresponding predicted AlphaFold structures. AlphaFold^27,28^ red and blue colors mark the degree of uncertainty of predicted structure, reflecting the degree of disorderness. The ELM predicted phosphosites are marked by red circle lollipops, sites predicted by ELM^33^ and confirmed using iProtein DB^34^ are marked by filled red circles. (**a**) NAC-α, (**b**) gNAC-α, (**c**) NAC-β, (**d**) gNAC-β, (**e**) uncNAC-β.

Studying the conformation of the ubiquitously expressed NAC from *Caenorhabditis elegans* revealed its remarkable flexibility ensuring its ability to bind various protein substrates, irrespective of whether they are folded or intrinsically disordered^24^. Discovering of the germline paralogs of both NAC subunits prompted us to compare the predicted conformational flexibility of their sequences with ubiquitous paralogs by assessing the IDRs outside the folded NAC domains known to handle αβ dimerization. The profiles of the disordered regions were obtained with the IsUnstruct program^25,26^, the order–disorder profiles of NAC containing polypeptides were predicted by AlphaFold^27,28^ (Fig. 2). Two additional programs, PONDR_FIT^29^ and IUPred2long^30^, yielded similar results thus demonstrating a good agreement for all three programs (Figure S1). Both ubiquitous and germinal α-subunits contain the folded NAC-domain and the ubiquitin-activated domain (UBA), which was recently shown to be the SRP interactor, stabilizing the co-binding of SRP and NAC on the signal-sequence displaying ribosomes^8^. The germinal α-subunit has an approximately three-fold increase in IDR length at the N-terminus compared with the ubiquitous paralog (Fig. 2a,b). The ubiquitous β-subunit carries a conserved motif at the N-terminus of the ribosome associated region being positively charged^1^ and the NAC dimerizing domain, while germinal paralog preserving this charged motif has a more than a twofold increase in the IDR length at the C terminus compared to the ubiquitous paralog (Fig. 2c,d). Thus, a significant difference in amino acid sequence patterns between the germinal and ubiquitous subunits lies in the extensions of germinal-specific IDR lengths (Fig. 2a–d). Notably, the positively charged sequence of the N terminus of the NAC-β subunit from *C. elegans* was recently shown to be responsible not only for ribosome binding but also, being unstructured, has the ribosome independent chaperone activity^31^.

**Figure 3:**
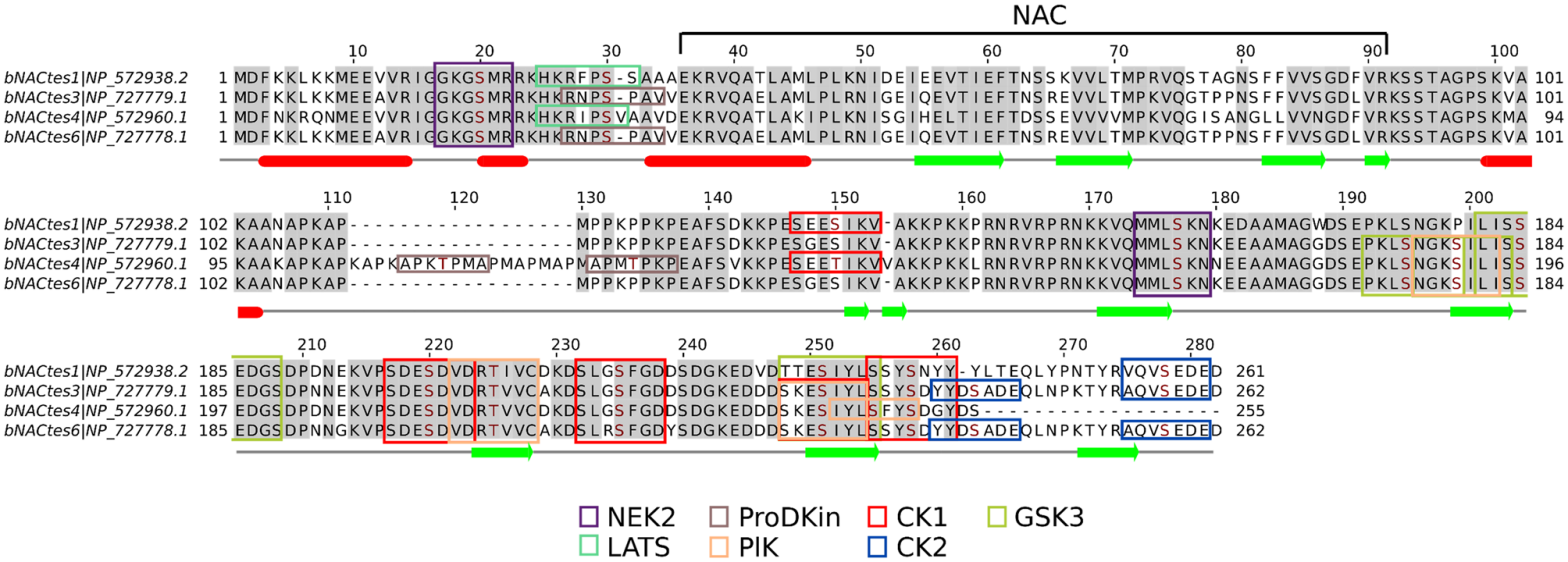
Predicted phosphosites in IDRs of the gNACβ subunits. Docking sites for various Ser-Thr protein kinases (CK1, CK2, NEK2, LATS, ProDKin, PIK and GSK3-Ser) are indicated; X-linked paralogs of *D. melanogaster* (Flybase.org, ELM Database^33,34^, α-helices and β-sheet regions are designated with red bars and green arrows, respectively.

Phosphorylation is regarded as one of the most common types of PTM in long (> 100 aa) IDRs^32^. We used ELM^33^ (eukaryotic linear motif resource for functional sites in proteins, http://elm.eu.org) and iProteinDB^34^ (integrative Database of Drosophila Post-translational modifications) to predict phosphosites and docking motifs for protein kinases along the gNAC-β and gNAC-α sequences compared with the ubiquitous paralogs (Fig. 2a–d), as well as for several distinct gNACβ paralogs (Figs. 2, 3). This analysis showed that IDRs in all analyzed proteins are predicted to have numerous putative phosphosites for *Drosophila* protein kinases (Fig. 2). We also traced the diversity of the predicted phosphosites (Fig. 3) by comparing four highly homologous copies of gNAC-β gene^9^. The gNAC-β (tes4) demonstrated the emergence of a phosphosite within the newly acquired amino acid stretch and the acquisition of the adjacent phosphosite. At the same time, two phosphosites were lost from the very gNAC-βtes4 C terminus, compared to the other three copies.

**Figure 4:**
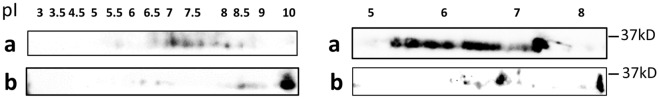
gNAC-β isoform spots from testis ribosomes. **(a)** Before and (**b**), after alkaline phosphatase treatment, showing spots vanishing or shifting to a region with a higher pI. Full blots in Expanded files 2, 3 in Supplementary.

Prior to directly demonstrating paralog phosphorylation, we performed a rough evaluation of the abundances of their transcripts using testis polyA mRNA NGS sequencing. We estimated the abundances of paralogous gNAC-β mRNAs, which turned out to be comparable, showing slightly higher abundances for the βNACtes3, 4 and 6 (the abundances of the corresponding mRNAs in TPM are as follows: tes4 (19.8), tes3 and tes6 (15.9), tes1 and 2 (equal, 7.5), tes 5, pseudogene, (1.0)). We checked whether gNAC proteins are phosphorylated in vivo. We performed 2D electrophoresis, using isoelectrofocusing in the first direction and revealed, by immunodetection, the extremely heterogeneous profile of the gNACβ proteins in the extracts of testis ribosomes. The observed pI range of the major spots of gNAC-β proteins is positioned in pI region 6.5–8, which is significantly lower than the theoretically predicted basic pI value 9.15–10 for the distinct NACβtes proteins (from Polypeptide Report of the Flybase). Correspondingly, the phosphatase treatment of the ribosomal extract resulted in the vanishing of spots from the more acidic region and drastic shifts of the gNAC-β spots for the distinct NAC-β germinal paralogs toward the alkaline pI values (Fig. 4a,b). This phosphatase-dependent diminishing of the spots concerns a more acidic pI region demonstrating the phosphorylation of gNAC-β subunits. The number of the detected spots (at least nine) is higher than the number of expressed paralogs (five), indicating dynamics in the alternatively phosphorylated states of the given germinal paralog.

### Functional crosstalk between the ubiquitous and germinal subunits of NAC

**Figure 5:**
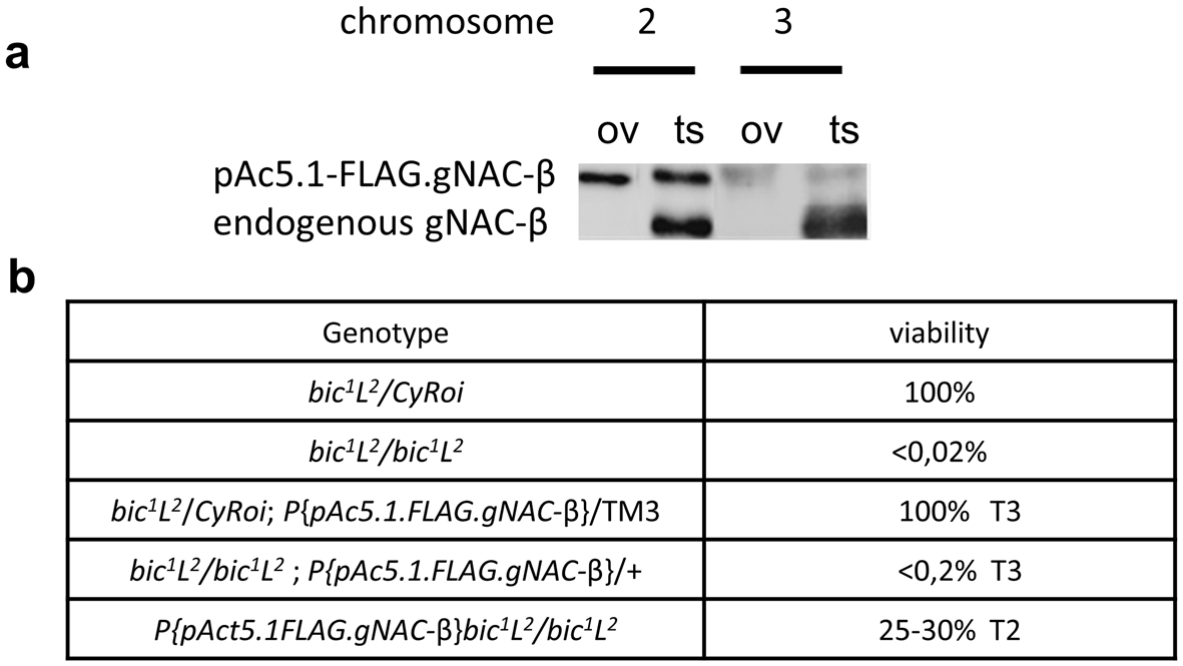
The lethal phenotype of the *bic1* mutation caused by the loss of the ubiquitously expressed NAC-β is rescued by ectopic gNAC-β expression. (**a**) Western blot expression analysis of FLAGgNAC-β transgenes (T2 and T3) inserted into chromosome 2 or 3, respectively. (**b**) Relative viability of the genotypes demonstrating transgene T2-induced suppression effect. Full blot for (**a**) in Expanded file 4 in Supplementary.

**Figure 6:**
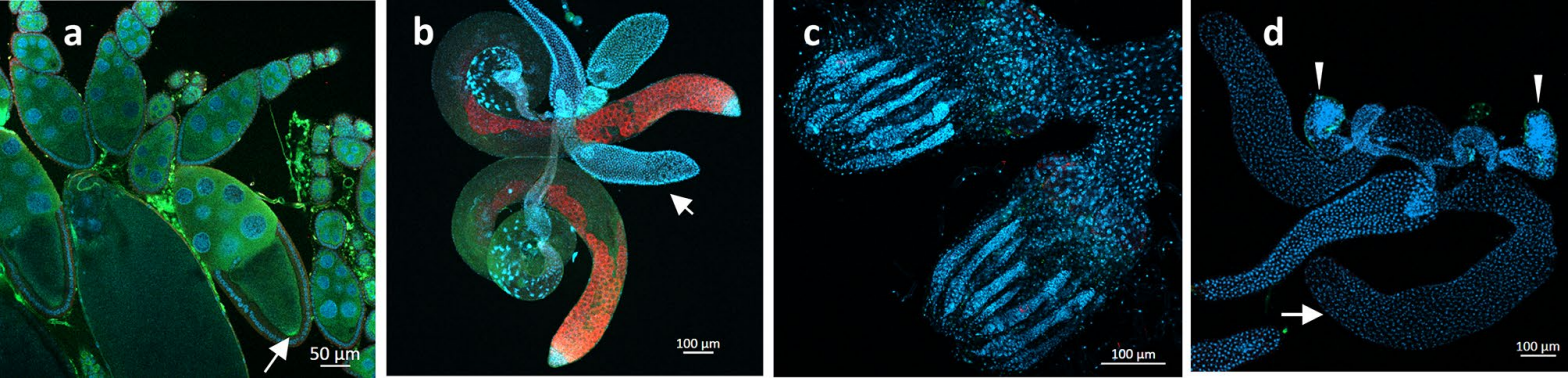
Germinal-specific siRNAi simultaneous knockdowns of both germinal and ubiquitous NACα disrupt the development of testis and oogenesis leading to complete infertility. DNA is stained with DAPI (blue), gNACβ (red), VASA (green). (**a**) Normal ovaries, the arrow indicates stage 9 of oogenesis and VASA expression in the nurse cells and posterior oocyte pole. (**b**) Normal testis, gNACβ immunostaining of spermatocytes, unstaining of somatic accessory glands (arrow) and seminal vesicles. **(c**,**d)** Adult ovaries and testes, images of double germinal knockdowns of germinal *(gNACα*) and ubiquitous *(ubNACα) NACα* genes using *nos-Gal4* > *UAS-gNACα-ubNACα-RNAi. *(**c**) The image of double germline knockdowns in ovaries showing DAPI stained somatic residues of elongated ovarioles lacking germinal cells. (**d**) Elimination of germinal cells and testes, the arrow points to the accessory gland, the arrowhead points to testis somatic remnant.

To determine whether the ubiquitous and germinal NAC subunits are functionally interchangeable, in particular, whether the NAC-α/gNAC-β chimeric heterodimer can perform at least some of the diverse functions of the ubiquitous αβNAC, we evaluated the ability of the ectopic expression of gNAC-β subunit to rescue the lethal mutation of the *bic*^20^, which encodes the ubiquitously expressed NAC-β subunit. Fly strains with the genomic insertions of transgene encoding gNAC-β driven by the actin5C promoter were generated and checked whether these transgenes are able to suppress the lethal effect of the null *bic*^*1*^ gene mutation^20^ (Fig. 5). The transgene insertions into chromosomes 2 (T2) and 3 (T3) were shown to provide high and medium levels of gNAC-β expression, respectively, as assessed by WB analysis (Fig. 5a). The T3 showed no visible suppressive effect (Fig. 5b), while T2 ensured the survival of up to 30% of fertile individuals. We put forward that gNAC-β protein carrying the additionally acquired C terminus IDR sequence is able to perform some functions of the ubiquitous NAC-β subunit in the chimeric NAC-α gNAC-β heterodimer. We also identified the role of the ubiquitous NAC-α subunit in determining gonad development and maintaining fertility. The separate germinal RNAi KD of the ubiquitous *NAC-α* or germinal *NAC-α* exert no visual phenotypes, while combination of both KDs led to a strong disturbance of gonad development and infertility (Fig. 6c,d, see legend) without affecting fly viability. The obtained results correlate well with the STRING database presenting protein–protein interaction network encompassing the NAC domain carrying proteins.

### The germinal and ubiquitous NAC paralogs in the genomes of related species

**Figure 7:**
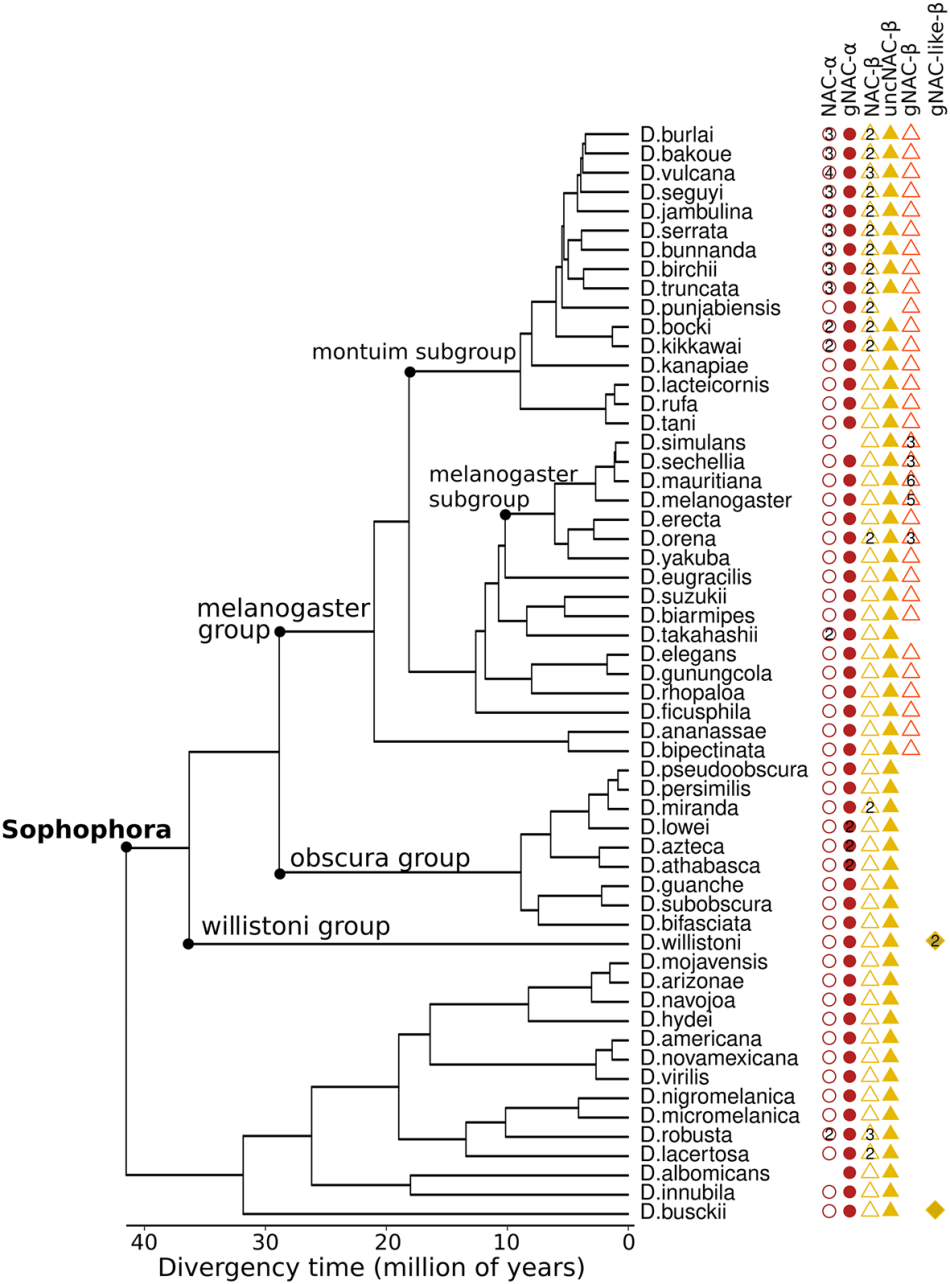
Phylogenetic tree of the *Drosophila* genus. The lack of some species (*mayri, watanabei, leontia, asahinai, pectinifera, obscura, montana, malanica and grimshawi*) on this tree is rather the result of the unfinished assembly of the corresponding genomes preventing the detection of the NAC domain sequences. The number of paralogs is shown within shapes.

The evolutionary origins of paralogous ubiquitous and germinal pairs of NAC proteins merit attention not only because germinal paralogs acquire IDRs but also because there is a specific «functional crosstalk» between the germinal and ubiquitous paralogs. The latter contributes to the ongoing debates concerning the evolution of paralogs, especially those capable of forming heterodimers^35^. We constructed the phylogenetic tree of 69 *Drosophilidae* species whose genomic assemblies were downloaded from the NCBI. The assemblies were subjected to BUSCO analysis^36^ to identify universal single copy orthologs followed by their multiple alignments and the construction of the phylogenetic tree of *Drosophila* species. The generated phylogenetic tree (Fig. 7) is in good agreement with those previously reported (e.g. «*Drosophila* 25 Species Phylogeny» 2017 Dataset posted on 28.09.2017). We found that the genomes of all mentioned species from the *Sophophora* subgenus, diverged from a common ancestor ~ 27 Mya, encode both the ubiquitously and germinal-expressed NAC-α as well as the ubiquitous NAC-β genes, whereas the amplified *gNAC-β* genes are found in the *melanogaster* group genomes*,* but not in *obscura* group.

**Figure 8:**
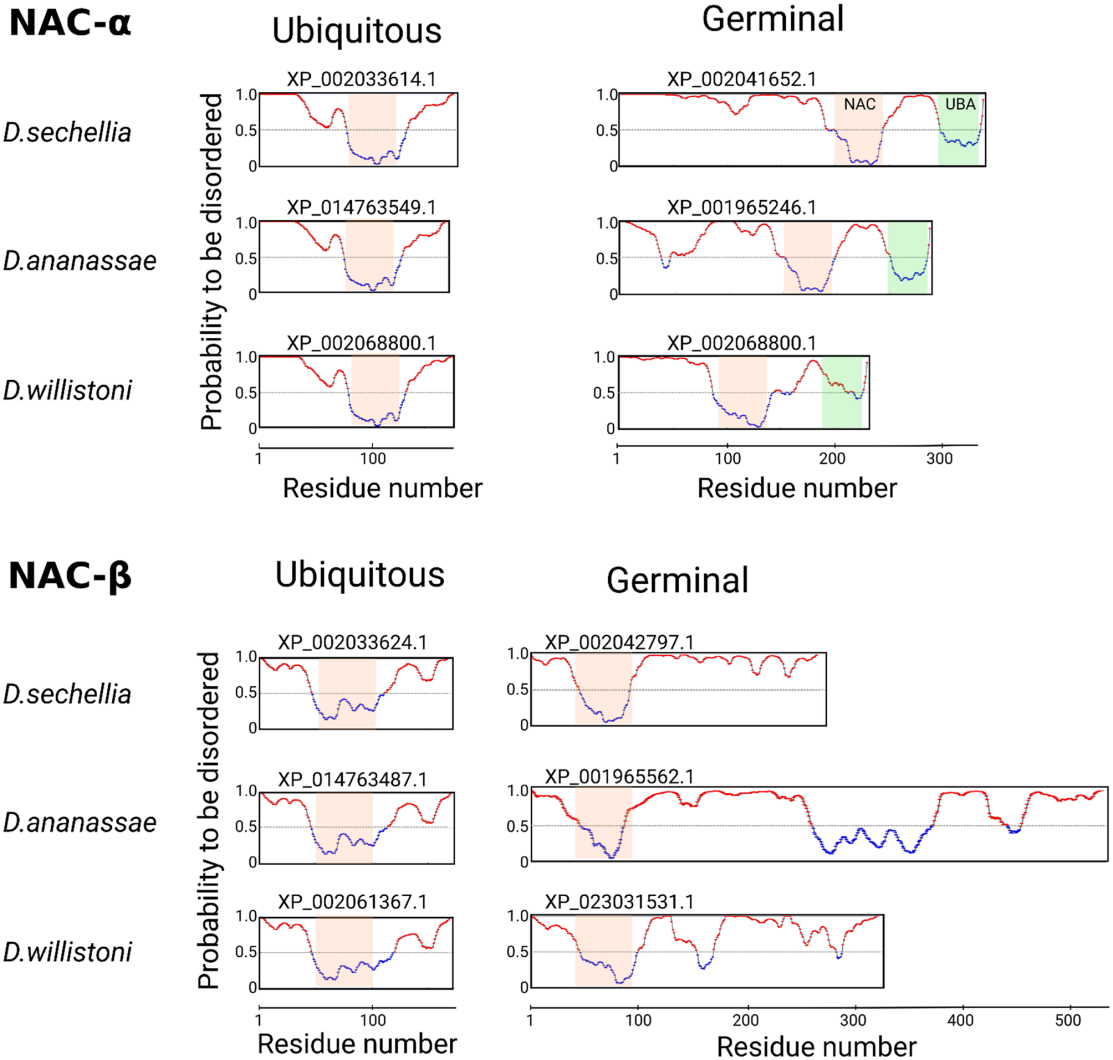
Comparison of order–disorder profiles of ubiquitous and germinal NACα and NACβ orthologs. Disordered and folded regions are marked with red and blue colors.

Comparison of the order–disorder patterns of the orthologous NAC-α and NAC-β subunits for three selected species related to three different *Drosophila* clades presented in the phylogenetic tree is shown on Fig. 8. The evolutionary unchanged patterns of the ubiquitously expressed orthologs, including short disordered parts are evident, in contrast to the high level of diversification, including significant lengths variations, detected for the germinal counterparts. A separate observation concerns the preserved UBA-like domains keeping 64–70% identities to the *D*. *melanogaster* paralog in the NAC-α germinal orthologs. However, the UBA domain is shown to be lost in the ubiquitously expressed orthologs, positioning the question of this domain’s specific function in the germline of several species.

We also identified the *gNAC-β-like* genes carrying extended IDRs in the genomes of earlier branched-off species, *D. willistoni* and *D. busckii*. These IDRs, having comparable lengths to those in *D. melanogaster*, are characterized by more orthologous divergencies than the NAC domain sequences (Supplementary Figure S2). Interestingly, despite a high level of the orthologous polymorphism, the distribution of S/T locations in the region of the predicted phosphosites was shown to be positionally conserved when comparing IDR orthologous sequences from *D. willistoni* and *D. melanogaster*. Comparing the corresponding sequences encompassing ~ 50 amino acid stretches reveales conservative locations of six S/T positions marked by a red line with crosses (Supplementary Figure S2) intermingled with the rest of multiple amino acid substitutions. Detecting conservative positions of putative functional phosphosites in orthologous IDR sequences is in accord with the notion that IDRs are conserved across orthologs in the vast majority of cases^37^ as well as the recent report of conservative RNA binding domains positions in the orthologous IDRs, including the *Drosophila* and human pairs^38^. In the orthologous pair of IDR sequences of *D. willistoni* and *D.* *busckii,* in spite of a positional shift of serine-enriched cluster we also identified three conservative serine positions (Supplementary Figure S2, red circles).

The numbers of ubiquitous NAC-α and NAC-β copies were shown to correlate with each other in most species belonging to the *montium* subgroup of the *melanogaster* group (Fig. 7). This observation is consistent with the earlier report on the production of equimolecular α- and β-subunits in yeast cells^36,39^. Notably, the correspondence between gene numbers of the germinal α- and β-subunits is lost in several species from the *melanogaster* group, which are characterized by the amplification of the gNAC-β genes (Fig. 7).

The phylogenetic analysis of NAC homologs revealed that besides gNAC-β and ubiquitous NAC-β there is a third large family of NAC-β proteins (Supplementary Figure S3), named here as uncNAC*-*β (*unc*haracterized, *CG11835*, Flybase.org). Almost all species, except *D. punjabiensis* (Fig. 7), contain a single copy of *uncNAC-β* (*melanogaster* gene structure is presented in Fig. 2) encoding the NAC domain followed by about 800 amino acid residues of an extremely extended IDR. Comparing the orthologous IDRs revealed the clusters of conservative proline-dependent protein kinase phosphosites concentrated at the C-termini of uncNAC-β proteins (Supplementary Figure S3). Although the putative partners of uncNAC-β remain to be elucidated, we may suggest its heterodimerization with NAC-α subunits is capable of transferring some functionally important properties of IDRs to the newly formed heterodimers.

The cross-species alignment (Supplementary Figure S4, DataSet S1) showed that the ubiquitous NAC-α domains are highly conserved, while the gNAC-α domains diverged slightly faster (the average amino acid identity per column for multiple alignments of NAC domains was 80% and 75.6% for the ubiquitous and gNAC-α, respectively). The same feature is even more pronounced for the orthologous pairs of the NAC-β ubiquitous and germinal counterparts (the average identity per column in the multiple alignments of NAC domains is 69.4% and 38.8% for the ubiquitous and germline NAC-β domains, respectively). These values for the multiple alignments of the overall IDR sequences for gNAC-β are 8%, while the distinct regions within IDR reach the average identity of 50% implying a significant conservation. Noteworthy, some species subgroups from *Sophophora* subgenus have several highly similar paralogs of the αNAC and/or βNAC subfamilies.

## Discussion

Earlier, we have described five copies of testes-expressed highly homologous genes encoding β-subunits of ribosome associated NAC in the *D*. *melanogaster* genome^9,10,18^. These gene copies are localized in the distinct 12E region in the X-chromosome and are designated in Flybase as *betaNACtes1*, and two adjacent pairs of genes, *betaNACtes* 2,6 and *betaNACtes* 3,4^10^*.* To the best of our knowledge, we first detected the tissue specific subunit of NAC that is thought to be involved in protein homeostasis^10^ in multicellular eukaryotes^2,3^. Here, we discovered the germinal NAC-αβ paralog, whose α-subunit was shown by in situ hybridization to be expressed exclusively in the germline during the early embryonic development. In this paper, we also characterize the expression of heterodimeric αβNAC encoded by the corresponding plasmids carrying the tagged *gNAC-α* and *gNAC-β* genes in cell culture. The expression of the *gNAC-β* genes was traced in germ cells during the early embryonic development of *D. melanogaster,* allowing us to use the designation “gNACs” for the germinal specific heterodimeric NACs. Earlier, we failed to observe the negative effect of siRNA *βNACtes* KD on the fertility in testes, possibly due to an incomplete depletion of the mRNAs of these amplified genes^9^*.* Identifying a unique gene for the gNAC-α subunit, presented in this paper, opens a way to eliminate gNAC-α protein activity using CRISPR-Cas9 gene editing to trace the putative negative effect on fertility.

Comparing the amino acid sequence patterns of the ubiquitous and germinal NAC subunits revealed the significantly extended IDRs at the C- and N-termini of the β- and α-subunits of gNAC, respectively. The burst of interest to IDRs stems from a large amount of the recently accumulated data demonstrating the wide functional role of IDRs in protein–protein interactions and the formation of biocondensates^40,41^. IDRs are protein unfolded parts providing protein–protein interactions^42^, which are considered to be necessary for the functionality of the ubiquitous NAC and its chaperone activity in *C. elegans*^31^. We suggest that germinal-specific evolutionary acquired IDR sequences participate in sophisticated germline protein-specific interactions. Here we detected the presence of phosphorylated isoforms of germinal NAC-β subunits. We also predicted the excess of phosphorylation sites at the extended IDRs of gNAC subunits comparing to the ubiquitous paralogs. Given the crucial role of NAC with its flexible adaptor sites ensuring interactions between the nascent polypeptide and the components of the protein sorting machinery^1,2^ and functional conformational mobility of phosphorylated IDRs^43,44^, further in-depth experimental studies of NAC phosphorylation will be necessary to discern the peculiarities of their functions in somatic and germinal cells. However, associating these IDR regions with specific biological and biochemical functions based on their amino acid sequences and putative PTM sites^45^ remains a challenge.

Detecting specific NAC in the germline is of particular interest, considering some peculiarities of protein synthesis regulation in the germline development, associated with proliferation and differentiation programs^46^. We suggest that the germinal NAC subunits, carrying the extended flexible amino acid sequences easily accessible for PTM, can be used in the germline proteostasis regulation. The recent in vitro study of human βNAC function using reticulocyte rabbit ribosomes revealed a novel NAC function as a component of the ternary complex «NAC-ribosome nascent chain complex-SRP with SR-receptor» located at the ribosomal exit site^3,47^. The authors found ubiquitous NAC to selectively bias the flexible conformational landscape of multicomponent SRP to associate with its receptor during the cotranslational targeting of the nascent polypeptide carrying the N-terminal signal to ER. Thus, a more active and broader role of the ubiquitous NAC in proteosynthesis as an allosteric regulator of SRP^31^ emphasizes that specific interactions involving IDRs of both gNAC subunits can be functional in the germline.

In a recent work devoted to coordinated co-translational regulation of ribosomal proteins in yeasts^48^, co-translational chaperones responsible for the folding of these nascent ribosomal proteins, were also shown to stabilize the corresponding translated RNAs. At the same time, NAC protein competed with the dedicated chaperones for the nascent protein in the ribosome tunnel, which is accompanied by mRNA degradation due to the NAC ability to recruit the mRNA deadenylation complex to normalize ribosomal proteins production. Thus, NAC is also involved in regulating the amount of translated mRNA. These processes orchestrated by a thoroughly regulated intermolecular flexible partner interactions, also well known to be attributed to the granule formations in the germlines of multicellular eukaryotes.

Here we have also found that the lethal effect of the lack of ubiquitous NAC-β subunit is rescued by the ectopically expressed germinal paralog carrying IDRs. This observation does not mean that the ubiquitous heterodimer could be replaced with a germinal paralog, since the effectiveness of the chimeric gNACβ-NAC-α heterodimer in maintaining protein homeostasis has not been evaluated. The observed suppression indicates only a certain level of functional flexibility of the heterodimer subunits. Nevertheless, here we found a functional crosstalk between the ubiquitous and germinal paralogs of both α and β subunits of NAC. This type of crosstalk is known to be accompanied by a well-known asymmetric evolution (subfunctionalization) of paralogs^49^ without violating their potential interchangeability. We suppose that this functional crosstalk may be beneficial for maintaining the reproduction system based on germinal cell proteome robustness. This type of paralogous interactions was shown to be especially relevant for those paralogs that can form heterodimers^50^, as is in the case of NAC paralog subunits.

The switch-like decisions during germ cell development are proposed to be closely related to the formation of large protein complexes, including large granules, also known as membraneless organelles (for review^51,52^). In this regard, new ideas about the cotranslational assembly of multisubunit protein complexes with a chaperone based on rigorous experiments are of particular interest^2,53–55^. The authors propose that the translations of subunit complexes are spatially confined. These ideas are discussed in terms of compartmentalized translation principles with regulatory implications in development^56,57^. It would be fruitful to extend these concepts to describing the complex processes of granule formation in the germ plasm compartment of a mature oocyte where gNAC is localized accord to our preliminary experiments. Identifying gNAC still only open up an opportunity for finding experimental approaches to explore its function.

## Methods

### Fly stocks and crosses

*Batumi* line laboratory stock was used in biochemical experiments and immunostaining. To generate recombinant chromosome 2 carrying the *bic*^*1*^ lethal allele and FLAG.gNACβ transgene, the *y*^*1*^w*; Eney-whited700-pAc5.1.FLAG.gNAC-β.*RetMI07200*/*CyRoi* females were crossed to *bic*^*1*^*L*^*2*^*/CyRoi* males and the F1 *Eney-whited700-pAc5.1.FLAG.* g*NAC-*β.*RetMI07200/bic*^*1*^*L*^*2*^ females were crossed with *yw*^*67c23*^, +/+ males. The male progeny of this cross with recombinant chromosome (marked *whited700* and *L*^*2*^) *Eney-whited700-pAc5.1.FLAG.gNAC-*β.*RetMI07200 bic*^*1*^*L*^*2*^/+ were collected and individually crossed to *yw*^*67c23*^, +/*CyRoi* females. Males *Eney-whited700*-*pAc5.1.FLAG.gNAC-*β.*RetMI07200 bic*^*1*^*L*^*2*^*/CyRoi* were crossed with *bic*^*1*^*L*^*2*^*/CyRoi* females and the viable *Eney-whited700-pAc5.1.FLAG.gNAC-*β.*RetMI07200 bic*^*1*^*L*^*2*^*/bic*^*1*^*L*^*2*^ individuals (lacking *CyRoi* and carrying the *whited700* and *L* markers) were traced/selected to evaluate the suppression effect of the *bic*^1^ lethality.

Germinal knockdowns: *nos-Gal4* > *UAS-gNACα-RNAi* and *nos-Gal4* > *UAS-ubNACα-RNAi* (VDRC) were performed separately or simultaneously using *nos-Gal4* > *UAS-gNACα-ubNACα-RNAi.* The driver line was obtained from Bloomington Stock #25751, *P{UAS-Dcr-2,D}1,w*^*1118*^; *P{GAL4-nos,NGT}40*. RNAi lines include stocks from VDRC: # 102621 (gNACα (*CG4415*)), and # 36017 (ubiquitous NACα (*CG8759*)).

### Constructs of plasmids

RT-PCR products of gNAC-β and gNAC-α ORFs were inserted into pAc5.1. FLAG or pAc5.1.HA carrying plasmids. Oligos for plasmid insertions:

pAc5.1.FLAG.gNAC-β (CG18313):

Xho1-5′tataCTCGAGACAATGGATTTCAACAAGCGACAG.

Apa1-5′tataGGGCCCCTAATCTTCGTCCTCGGAGACCT;

pAc5.1.HA.gNAC-α (CG4415):

Kpn1-5′tataGGTACCTTCCTCAAGATGGGTAAGAAGCAGA.

Xho1-5′tataCTCGAGGTTGTCGTTCTTCAGCAGCGC.

### Generation of transgenic lines expressing FLAG.gNACβ protein under pAc5.1 promoter

Transgenic strains carrying construct *attBs-Eney-whited700-pAc5.1.FLAG.gNAC-β-attBsrev-pSK* = *aeca* were generated by *phiC31*-mediated site-specific integration at the MiMIC site^58–60^ in the *Ret* gene of chromosome 2 (Bloomington #43099, y1w*; MiRetMI07200/SM6a) and at the site (Bloomington #24862, *yM{RFP[3xP3.PB] GFP[E.3xP3]*=vas-int.Dm}ZH-2A w[*]; PBac{y[+*]-attP-9A}VK00005*) of chromosome 3. The *vas-dPhiC31* strain bearing the *phiC31* gene under the control of the *vasa* gene promoter on the X chromosome was used as an integrase source^59^. The germ-line transformation of the embryos was performed according to standard protocol^61^ with the approximately 40% efficiency of integration.

### Generation of antibodies against germinal NACβ subunit

Here we generated rabbit anti His-tagged germinal NAC-β subunit antibodies using antigen sample as described earlier^10^. Antibodies were purified by antigen affinity chromatography using Thermo Scientific AminoLink Plus Coupling Resin according to the manufacturer’s protocol (Thermo Fisher Scientific). The generated antiserum was shown to recognize exclusively the germ cells in testes and early embryos and was used in Western-blot analysis (dilution 1:1000).

### Cell culture transfection, immunoprecipitation and western-blot analysis

Transient transfections of S2 cells were performed with the help of FuGENE^®^ HD Transfection Reagent (Promega# E2311) according to the manufacturer’s instructions. 3–4 days after the transfection the cells were harvested and subjected to immunostaining. For immunoprecipitation, anti-HA Magnetic Beads (#88836 Thermo Scientific) or anti-FLAG M2 Magnetic Beads (M8823 Sigma) were used. Protein samples (S2 cells or fly extracts) were applied to SDS-PAGE, transferred onto PVDF membrane according to standard protocols. The blots were analyzed using antibodies in a dilution 1:1000 against germinal NACβ, monoclonal mouse anti-FLAG M2 (Sigma F3165) and monoclonal mouse anti HA-Tag antibodies (mAB#2367, Cell Signaling Tech). Alkaline-phosphatase-conjugated anti-rabbit or anti-mouse antibodies (Sigma) were used as secondary reagent at a dilution of 1:20,000. Blots were developed using the Immun-Star AP detection system (Bio-Rad Laboratories) in accordance with the recommendations of the manufacturer, the signal was detected using the BioRad Chemi Doc MP Imaging System.

### Ribosome isolation and 2D resolution of ribosomal proteins from testes

250 pairs of frozen testes were homogenized in a Dounce homogenizer in 1.5 ml of buffer containing 25 mM Hepes [pH 7.6], 100 mM KCl, 5 mM MgCl_2_, 1 mM DTT, RNase inhibitor RiboLock (Thermo Scientific) at 40 units/ml, 0.015% digitonin, 1% NP-40, 0.5% sodium deoxycholate and 100 µg/ml cycloheximide. A protease inhibitor cocktail was used, as recommended by the company (Protease Inhibitor Cocktail Tablets, Roche). Cell fragments and mitochondria were removed by centrifugation at 12,000*g* at 4 °C for 20 min as described earlier^10^. 1.5 ml of post-mitochondrial extract were centrifuged for 1 h at 35,000 RPM (100000 g) in a rotor himac P50A3-0529, ultracentrifuge CP100-NX, at 4 °C. 2D electrophoresis was performed at the «Human Proteome» Collective Use Center of the V.N. Orekhovich Federal State Budgetary Scientific Institution Research Institute of Biomedical Chemistry.

For the first dimension, a ribosomal pellet or a protein precipitate after dephosphorylation was suspended in 250 µl of buffer (7 M urea, 2 M thiourea, 4% [w/v] CHAPS, 1% [w/v] DTT, 2% immobilized pH gradient [IPG] buffer [pH 3–10], protease and phosphatase inhibitor cocktails [Roche Diagnostics, Mannheim, Germany]), then clarified for 10 min at 10,000 g at 4 °C. 120 µl of clarified protein solution mixed with 30 µl of rehydration buffer (7 M urea, 2 M thiourea, 2% CHAPS, 0.3% DTT, 0.5% IPG buffer [pH 3–11 NL], and 0.001% bromophenol blue) were used to prepare the first-dimensional gel. 7-cm IPG gel strips (pH 3–10) were rehydrated passively for 10 h at 4 °C. IEF was conducted at 20 °C using Protean IEF Cell (“Bio-Rad”). Power supply was programmed in the gradient mode with voltages for four steps: first − 300 V (00.30 min), second-gradient 1000 V (00.30 min), third-gradient 5000 V (01.20 min), fourth and hold – 5000 V (00.25 min). Prior to the second dimension, the IPG gel strip was soaked in equilibration solution 950 mM Tris–HCL [pH 6.8], 6 M urea, 2% SDS, 30% glycerol) containing 1% DTT for 10 min. This process was followed by a 10-min incubation in the equilibration solution containing 5% iodacetamide. The IPG gel strip was then placed on top of the second-dimensional stab gel and sealed using 1 ml of molten agarose with 0.5% TGS electrode buffer (24 mM Tris [pH 8.3], 200 mM glycine, and 0.1% SDS). The second dimension SDS-PAGE was carried out using Hoefer miniVE vertical electrophoresis system [12% w/v gel concentration, 80 × 90 × 1 мм in size]. Precision Plus Protein Standards (BioRad) were used as a marker. Electrophoresis was performed at constant current (25 mA/gel) and 100 to 160 V for 1.5 h at room temperature. On completion of electrophoresis, the gel was rinsed in transfer buffer (48 mM Tris, 39 mM glycine, 0.037% SDS, and 20% methanol) and transferred to a PVDF membrane using a semidry transfer cell (Bio-Rad) following the manufacturer’s instructions.

Ribosome treatment by calf intestinal (CIP Sigma #4978) alkaline phosphatase was performed according to the protocol: a ribosomal pellet from 125 testes was suspended in 1 × CIP buffer (0.1 M NaCl, 0.05 M Tris–HCl pH 7.9, 0.01 M MgCl_2_, 1 mM DTT), 1 × Tm. Complete protease inhibitor mix (Roche), 20 units CIP, and the reaction mix were incubated for 120 min at 37 °C. Dephosphorylation was completed by adding cold 10% trichloroacetic acid. The precipitate was washed with acetone twice prior to isoelectrofocusing.

### Immunostaining

12–15-h embryos were collected, dechorionated in bleach (2%) for 3 min and after thorough rinsing with water were devitellinized in a 1:1 heptane/methanol mixture (− 20 °C) in a 1.5 ml microtube by gentle shaking. The devitellinized embryos were washed in the methanol phase, then rinsed 3 times with cold methanol. The embryos were stored at − 20 °C until immunostaining. For immunostaining, the embryos were gradually rehydrated with methanol-PBT (PBS with 0.1% Tween20), washed 3 times with PBTX (PBT with 0.3% Triton X100) and permeabilized in PBTX with 0.3% sodium deoxycholate (Sigma) for one hour. Then the embryos were washed three times in PBTX and blocked with PBTX containing 5% normal goat serum (NGS, Invitrogen) for 1 h. The embryos were first incubated with specific primary antibodies in PBTX containing 3% NGS overnight at + 4 °C and after washed 4 times in PBTX at room temperature, then incubated with secondary antibodies labeled with Alexa in a dark chamber overnight at + 4 °C. The embryos were mounted in Invitrogen SlowFade Gold Antifade reagent. The following primary antibodies were used: rabbit polyclonal anti-gNACβ (1:500), rat anti-VASA (1:200) (DSGB: AB_760351). The secondary antibodies were anti-rabbit IgG Alexa Fluor 546; anti-rat IgG Alexa Fluor 488 (Invitrogen, Thermo Fisher Scientific). Confocal microscopy was performed using Zeiss LSM 900.

Testes and ovaries of adult (1–2 day old) males and females were dissected in phosphate-buffered saline (PBS) at 4 °C, washed with PBT, fixed in 3.7% formaldehyde in PBT for 30 min at room temperature and then processed and immunostained like the embryos.

### Quantification of NACs mRNA abundances

The abundances of testis-specific NACs were calculated by Salmon Galaxy ver. 1.5.1 using R6.22 transcript fasta file and default settings. The source datasets and sequencing conditions are available in NCBI GEO, accession GSE101060.

### Phylogeny of 69 Drosophila species

To estimate the phylogeny of Drosophila species, the 192 RefSeq and GenBank genomic assemblies of 70 species were downloaded from the NCBI (Table S1). Each assembly was subjected to BUSCO v.4.1.4^36^ analysis to identify the universal single-copy orthologs from OrthoDB (-l dipteria_odb10). The genomic assembly of *Musca domestica* (GCF_000371365.1_Musca_domestica-2.0.2) was also analyzed by BUSCO for further use as the outgroup. 3119 universal single-copy BUSCO orthologs present in at least 90% of 192 assemblies and 69 genomic assemblies (one assembly per species) having at least 90% of 3119 single-copy orthologs were selected for the *Drosophilidae* phylogenetic analysis and the identification of NAC family proteins (Table S1). The concatenated multiple sequence alignments of the orthologous proteins with using MAFFT v7.471^62^ followed by alignment trimming with trimAl v.1.4^63^ (-gt 0.5) resulted in 710,094 amino acid columns that were used to estimate the maximum likelihood species phylogeny using RAxML v.8.0^64^ with the PROTGAMMAJTT model, rooted with the *Musca domestica*. We then used r8s^65^ to estimate branch lengths in millions of years with four calibration points^66^: 25–30 million of years (moy) for the common ancestor of *D. pseudoobscura* and *D. melanogaster*, 40 moy for the common ancestor of *D. virilis* and *D. melanogaster*, 6–15 moy for the common ancestor of *D. yakuba* and *D. melanogaster*, and 100 moy for the common ancestor of *D. melanogaster* and *Musca domestica*.

### The identification of NAC family proteins

The initial identification of NAC genes in *Drosophilidae* genomic assemblies was performed with *tblastn* v. 2.6.0^67^ (--e-value=10E−5) using NAC proteins from *D. melanogaster* as the queries. The hit regions extended with the additional 2000 bp on both sides were extracted from the genomes and the open reading frames were determined by AUGUSTUS v.3.1.3^68^. The identified open reading frames were confirmed as encoding NAC domains using InterProScan v.5.39^69^ (IPR016641 for αNAC and IPR039370 for βNAC). If the analyzed genome was already annotated by the NCBI (40 genomic assemblies), the accession number of the proteins was determined by *blastp* against the corresponding proteoms retrieved from the NCBI Protein database; otherwise (29 assemblies) the NAC protein was marked as ‘novel’. The multiple alignments of NAC proteins were carried out by the MAFFT v. 7.471^62^ (Data Set S1). The NAC domains of αNAC and βNAC were cut from the alignments and the positions including greater than ≥ 0.5 gaps were removed by trimAl, v.1.4^63^. Phylogenetic analysis of NAC domains was performed using the FastTree program^70^ with default parameters, with the WAG evolutionary model and the discrete *gamma* model with 20 rate categories. The tree structure was validated with bootstrap analysis (n = 100).

## Supplementary Information

Supplementary Information 1.

Supplementary Information 2.

Supplementary Information 3.

## Data Availability

The alignments used for the phylogeny are presented in Supplementary materials. NGS datasets are deposited in NCBI GEO, accession GSE101060. Materials (fly lines, etc.) from the paper are available upon request.
