# Supplementary material for: Extended disordered regions of ribosome-associated NAC proteins paralogs belong only to the germline in Drosophila melanogaster
Source: Sci Rep. 2022 Jul 1;12:11191. doi: 10.1038/s41598-022-15233-3 (PMC9249742; doi:10.1038/s41598-022-15233-3)
Supplement: Supplementary file 2 — Supplementary Information 2. [file 41598_2022_15233_MOESM2_ESM.pdf]

Extended disordered regions of ribosome-associated NAC proteins paralogs belong only to the germline in *Drosophila melanogaster*

Galina L. Kogan[1], Elena A. Mikhaleva[1], Oxana M. Olenkina[1], Sergei S. Ryazansky[1], Oxana V. Galzitskaya[2,3], Yuri A. Abramov[1], Toomas A. Leinsoo[1], Natalia V. Akulenko[1], Sergei A. Lavrov[1], Vladimir A. Gvozdev[1,*]

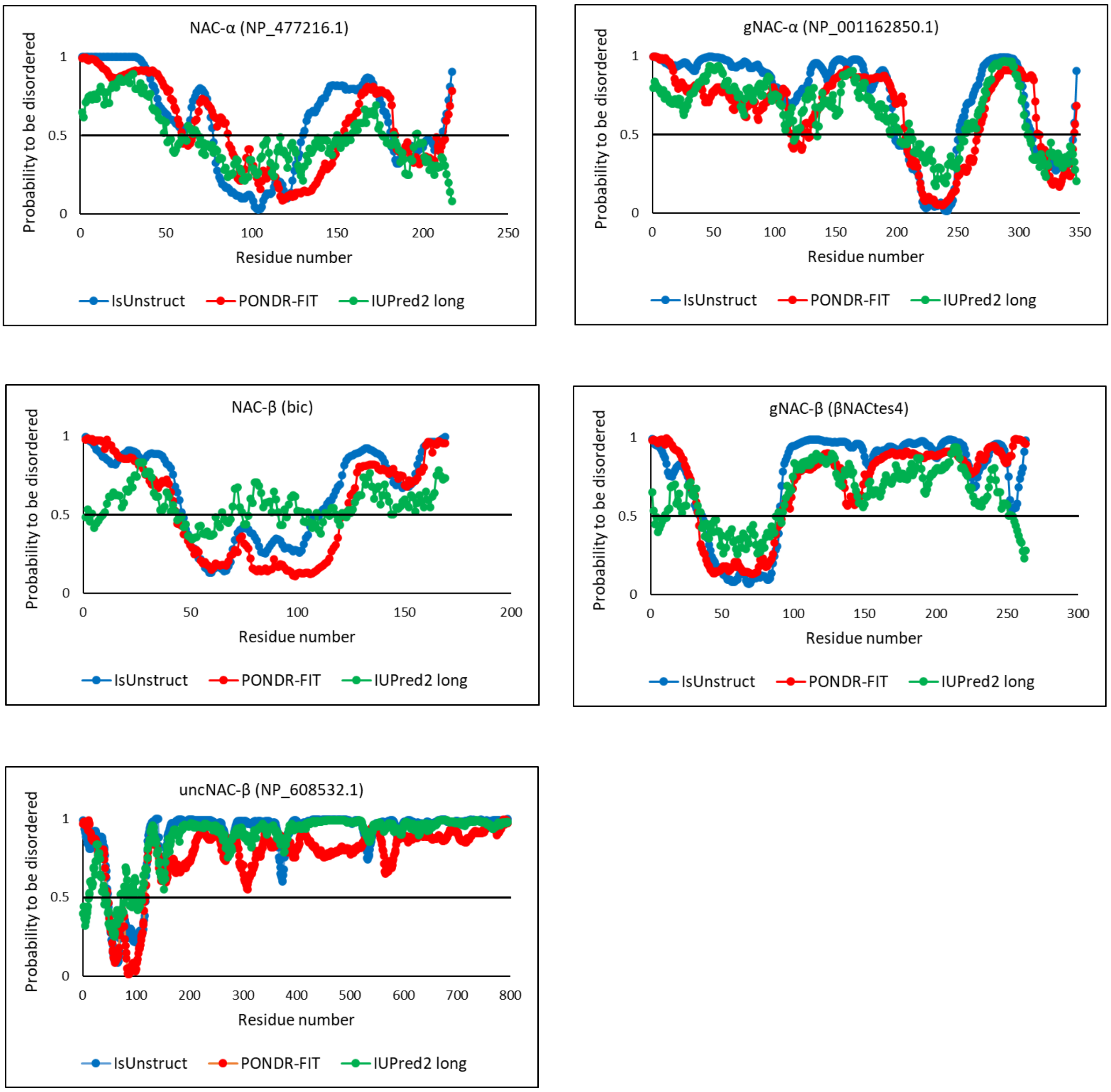

Figure S1. Comparison of predicted order-disorder profiles using IsUnstruct, PONDR-FIT, and IUPred2long programs.

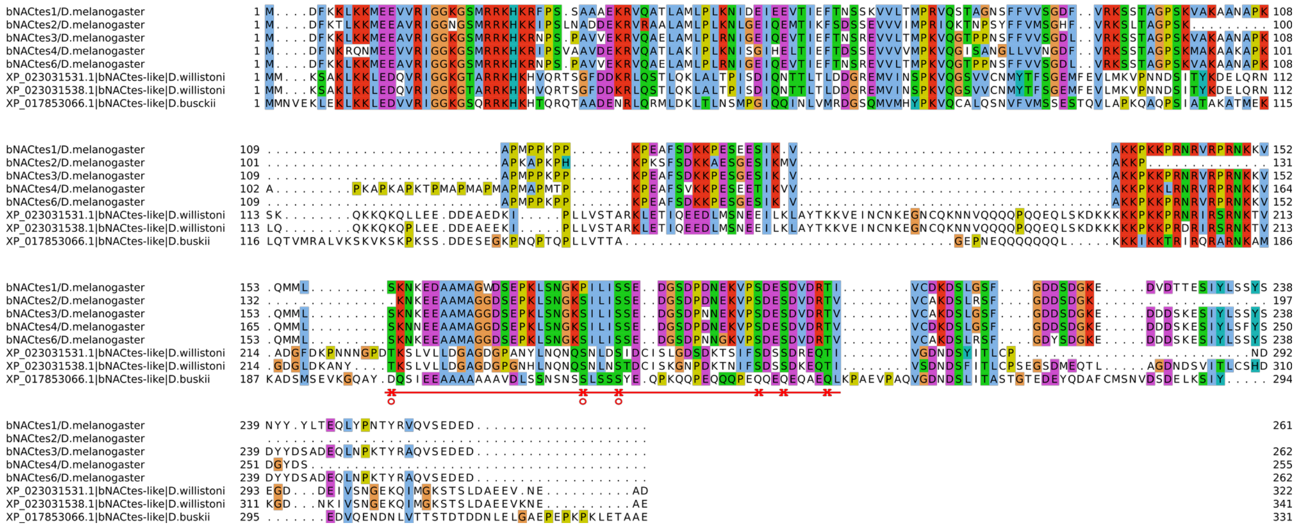

Figure S2. Alignments of the gNAC-βtes and orthologous gNAC-β-like proteins. The region with S/T phosphosites is underlined with a marked red line, crosses indicate the conservative Ser positions in *D. melanogaster* and *D. willistoni* IDR sequences; circles indicate conservative S positions between *D.willistoni* and *D.buskii* IDR sequences.

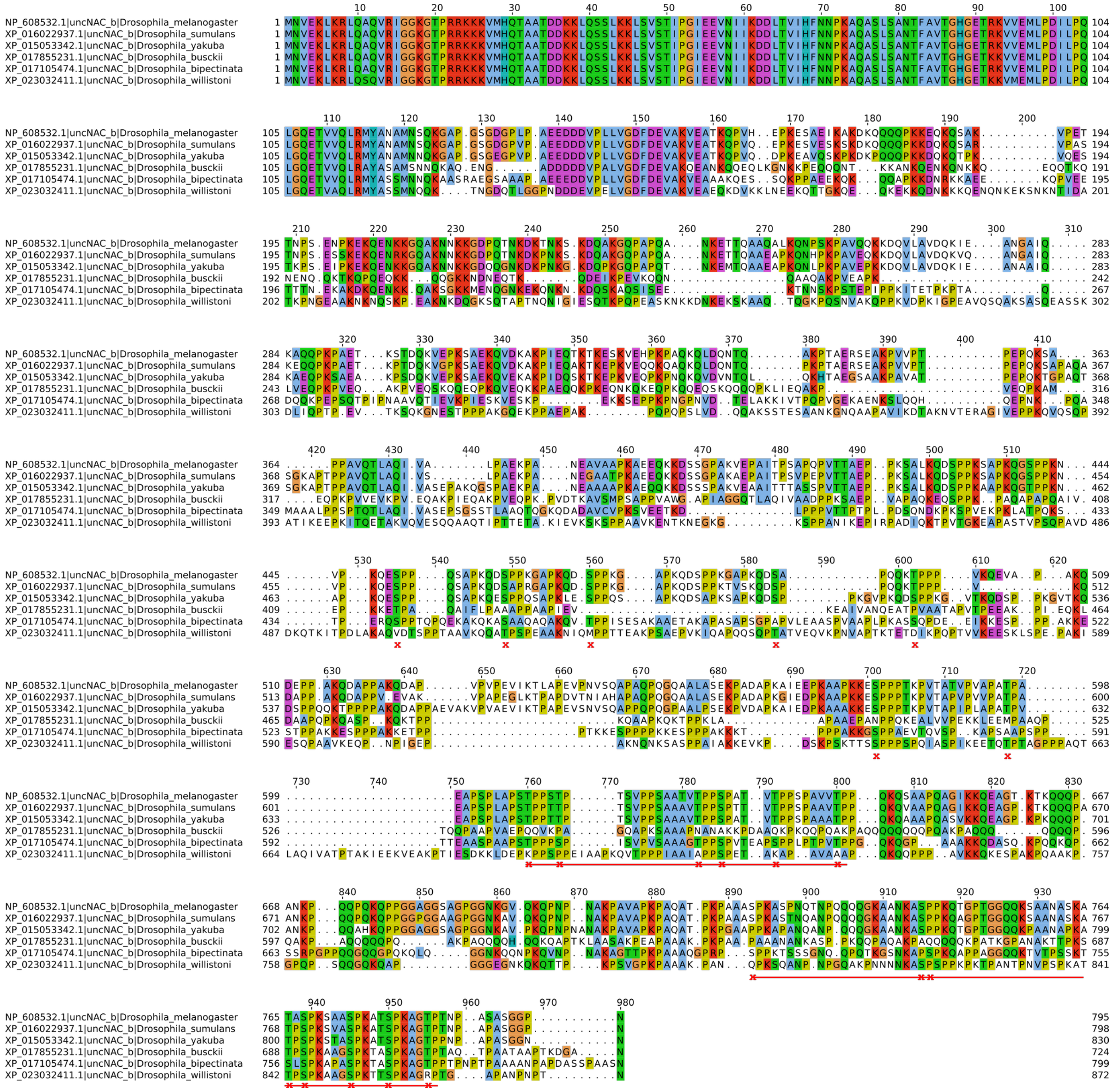

Figure S3. Conserved potential proline-dependent clustered phosphosites at the orthologous uncNAC-beta proteins with extended IDR- enriched C-ends; locations of the three phosphosite clusters are marked with a red line with crosses indicating phosphosites; other individual phosphosites are also marked with crosses.

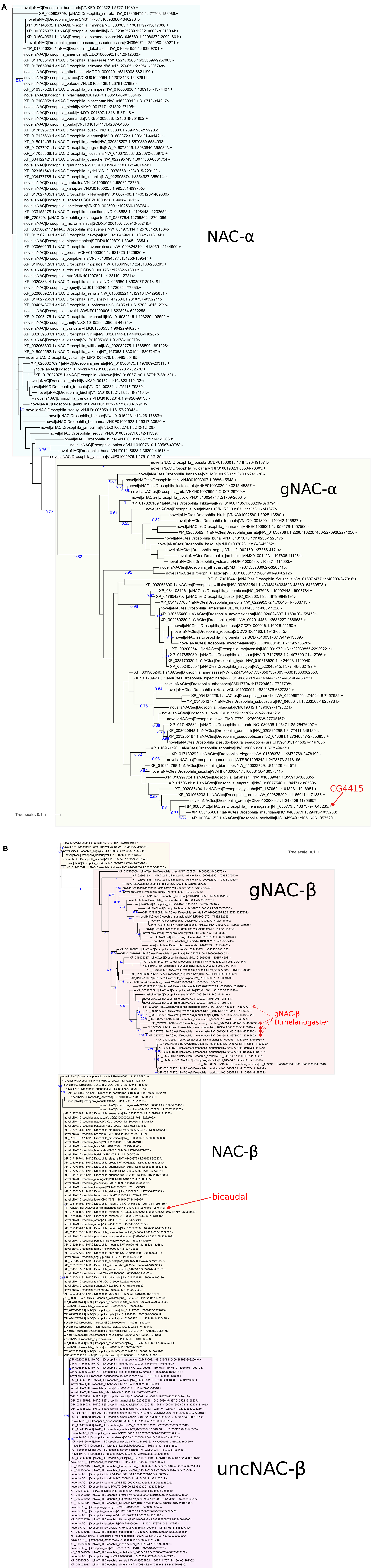

Figure S4. The phylogenetic trees for the NAC-α (**A**) and NAC-β (**B**) proteins of the *Drosophilidae* genomes. The species, accession number of protein and genomic coordinates of the gene are shown. The identified protein is marked as a "novel" if its accession number is non-available at the non-annotated genome. The blue integers indicate the bootstrap values (only values greater than 0.5 are shown).

Table S1. The genomic assemblies of *Drosophilidae* species used for the BUSCO analysis. For each genomic assembly the number and percentage of common unique single-copy orthologs are given. Only 69 genomic assemblies with >90% common unique single-copy orthologs were used for the phylogenetic analysis and the identification of NAC encoding genes.

Data Set S1. The multiple alignments of NAC-α, gNAC-α, NAC-β, gNAC-β and uncNAC-β proteins encoded in *Drosophilidae* genomes. For each protein the species, the accession number of protein and genomic coordinates of the gene are shown. If the accession number of the protein is not available for the non-annotated genomic assembly, then the protein is marked as “novel”.

Expanded File 1 to Figure 1b

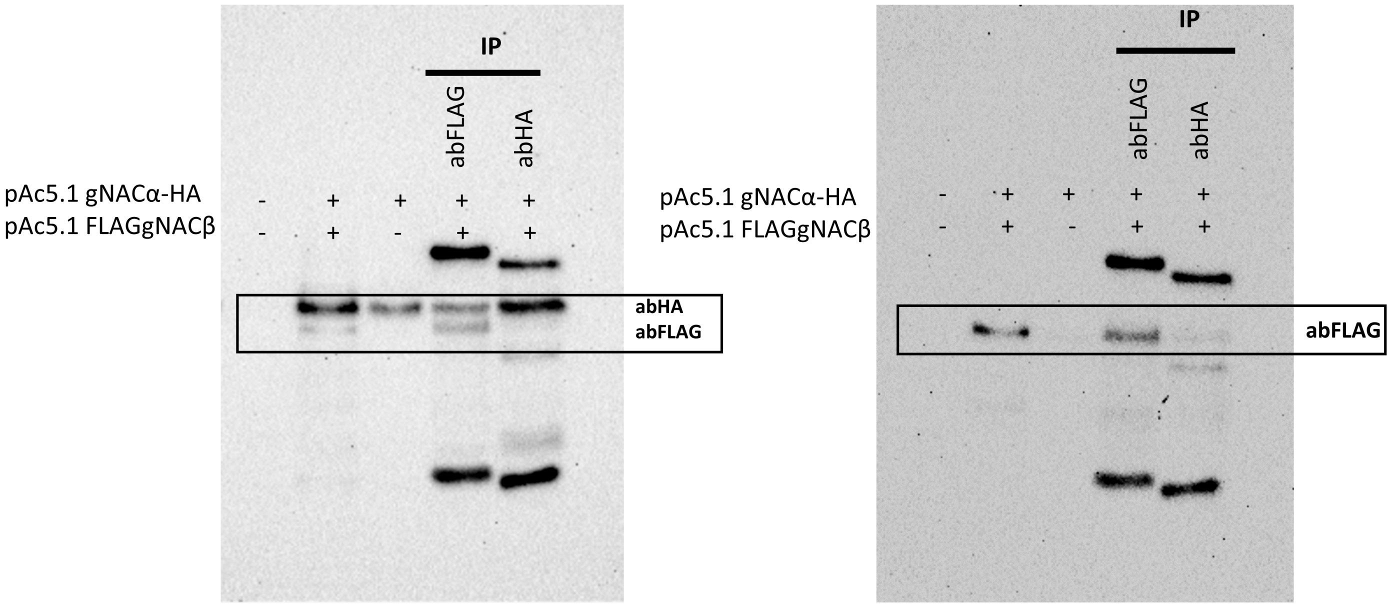

Transfecting S2 cells to express pAc5.1 gNAC-αHA and pAc5.FLAGNAC-β plasmids encoding NAC subunits

Expanded File 2 to Figure 4 (a,b) pI 3-10

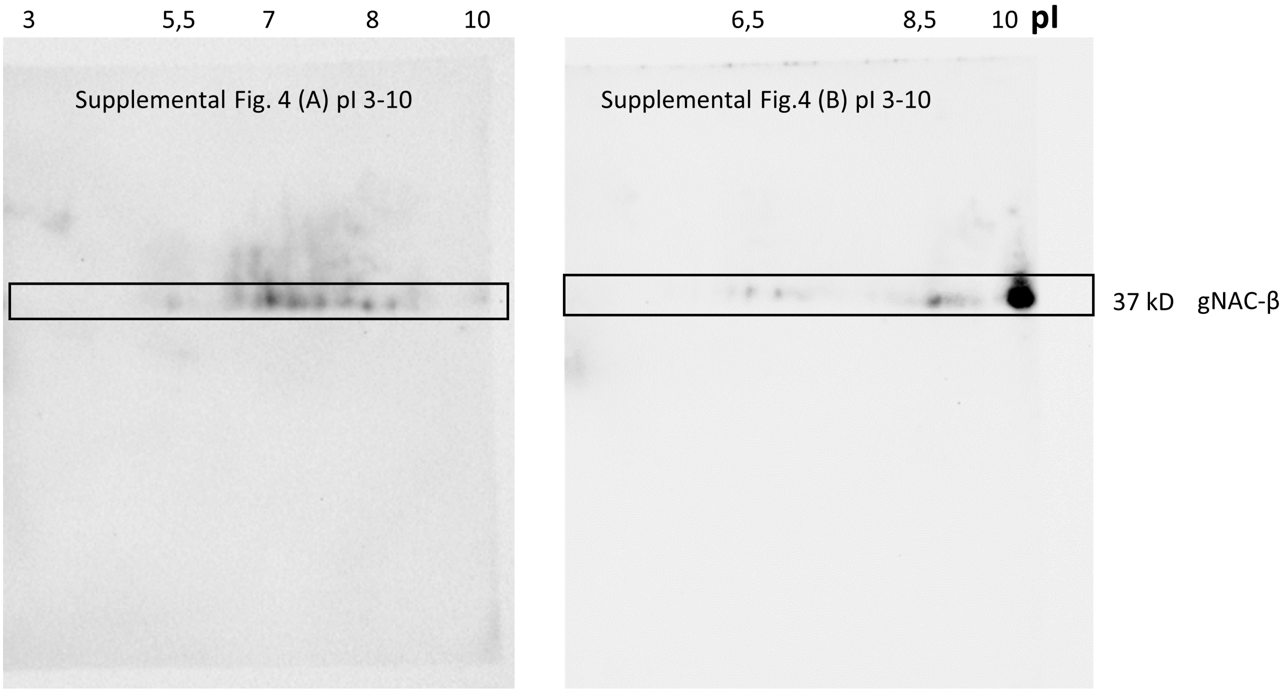

Expanded File 3 to Figure 4 (a,b) pI 5-8

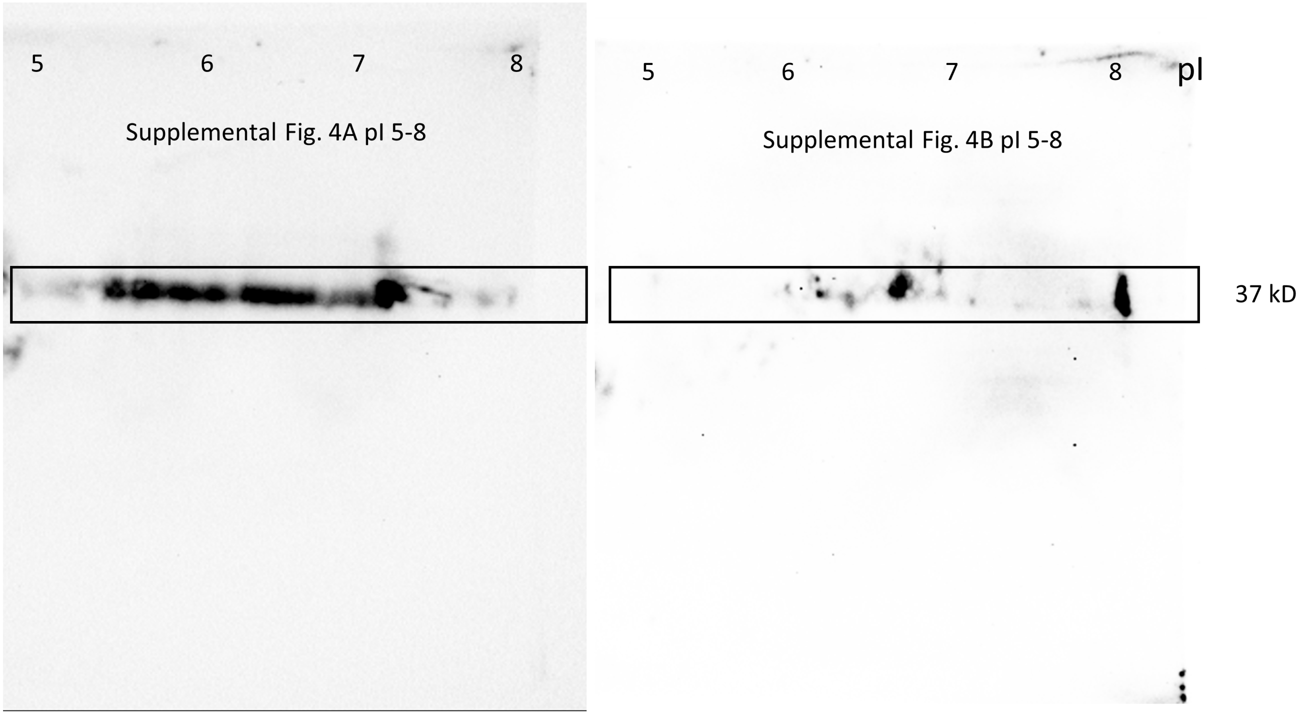

Expanded File 4 to Figure 5a

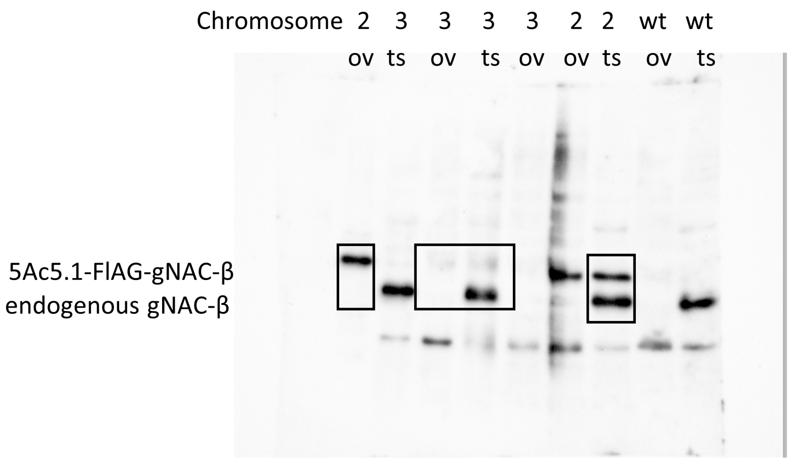

Ectopic (in ovary) gNAC-β expression from transgene on chromosome 2 is sighificantly higher then expression from the transgene on chromosome 3.
